# Bullous Systemic Lupus Erythematosus in a Male Child With Lupus Nephritis: A Rare Pediatric Presentation

**DOI:** 10.7759/cureus.106346

**Published:** 2026-04-02

**Authors:** Zara Saeed, Sulhera Khan, Sonia Golani, Nazish Shah, Mahwish Hassan

**Affiliations:** 1 Dermatology, Dr. Ruth K. M. Pfau, Civil Hospital Karachi, Dow University of Health Sciences, Karachi, PAK; 2 Internal Medicine, Jinnah Postgraduate Medical Centre, Karachi, PAK

**Keywords:** acr/eular criteria, bullous sle, bullous systemic lupus erythematosus (bsle), lupus nephritis, sle and lupus nephritis, systemic lupus erythematosus

## Abstract

Bullous systemic lupus erythematosus (BSLE) is a rare blistering manifestation of systemic lupus erythematosus (SLE), characterized by widespread tense bullae resulting from autoantibodies against type VII collagen. It is predominantly seen in women and is exceptionally rare in pediatric males. We report a case of a 14-year-old South Asian male with recurrent tense blisters over the trunk, face, extremities, palms, and mucosa, alongside systemic features including photosensitivity, arthralgia, oral ulcers, and significant renal involvement. Laboratory findings revealed proteinuria, hypoalbuminemia, low complement levels, and positive antinuclear antibodies. Skin biopsy revealed subepidermal blisters with neutrophilic infiltrates, and direct immunofluorescence (DIF) demonstrated linear IgG, IgA, IgM, and C3 deposition at the basement membrane. Renal biopsy revealed membranous glomerulonephritis along with positive DIF showing IgG and C3 deposition. Treatment with corticosteroids, dapsone, and mycophenolate mofetil led to significant clinical improvement. This case emphasizes the need for a high index of clinical suspicion in young males presenting with blistering dermatoses, as BSLE may serve as an early marker of active systemic disease, particularly lupus nephritis. BSLE is considered an important diagnostic red flag, especially in the pediatric population, because its appearance may signal active underlying systemic disease, as seen in our patient with lupus nephritis. Sudden development of widespread tense blisters may coincide with systemic disease flares. Early recognition and aggressive management with immunosuppressants, along with close renal monitoring and long-term follow-up, are essential to reduce irreversible cutaneous and renal complications.

## Introduction

Systemic lupus erythematosus (SLE) is an autoimmune, immune complex-mediated multisystem disorder that affects the skin, joints, cardiovascular and cerebrovascular systems, kidneys, and hematopoietic system [1]. Cutaneous involvement occurs in approximately 76% of SLE patients and can be classified into lupus-specific lesions, which exhibit histopathological features of interface dermatitis and include variants such as acute cutaneous lupus erythematosus, subacute cutaneous lupus erythematosus, and chronic cutaneous lupus erythematosus, whereas lupus nonspecific lesions are those that lack interface dermatitis and may include manifestations such as vasculitis, urticaria, livedo reticularis, and bullous eruptions [2]. Lupus-specific skin manifestations include the classic malar rash, discoid lupus erythematosus, lupus panniculitis, and chilblain lupus.

Bullous systemic lupus erythematosus (BSLE) is a rare nonspecific cutaneous manifestation of SLE, characterized by widespread blistering resulting from autoantibodies directed against type VII collagen [3]. Although cutaneous manifestations are common in SLE, BSLE accounts for only about 1% of cases [2]. The diagnosis of BSLE requires the presence of SLE as defined by the American College of Rheumatology criteria, an acute vesiculobullous eruption, a positive lupus band on direct immunofluorescence (DIF), detection of anti-type VII collagen antibodies, and exclusion of other vesiculobullous disorders [4].

Epidemiologically, BSLE predominantly affects women of childbearing age, particularly those of African descent [4]. Here, however, we report a case of a young South Asian boy presenting with classical features of BSLE.

## Case presentation

**Figure 1:**
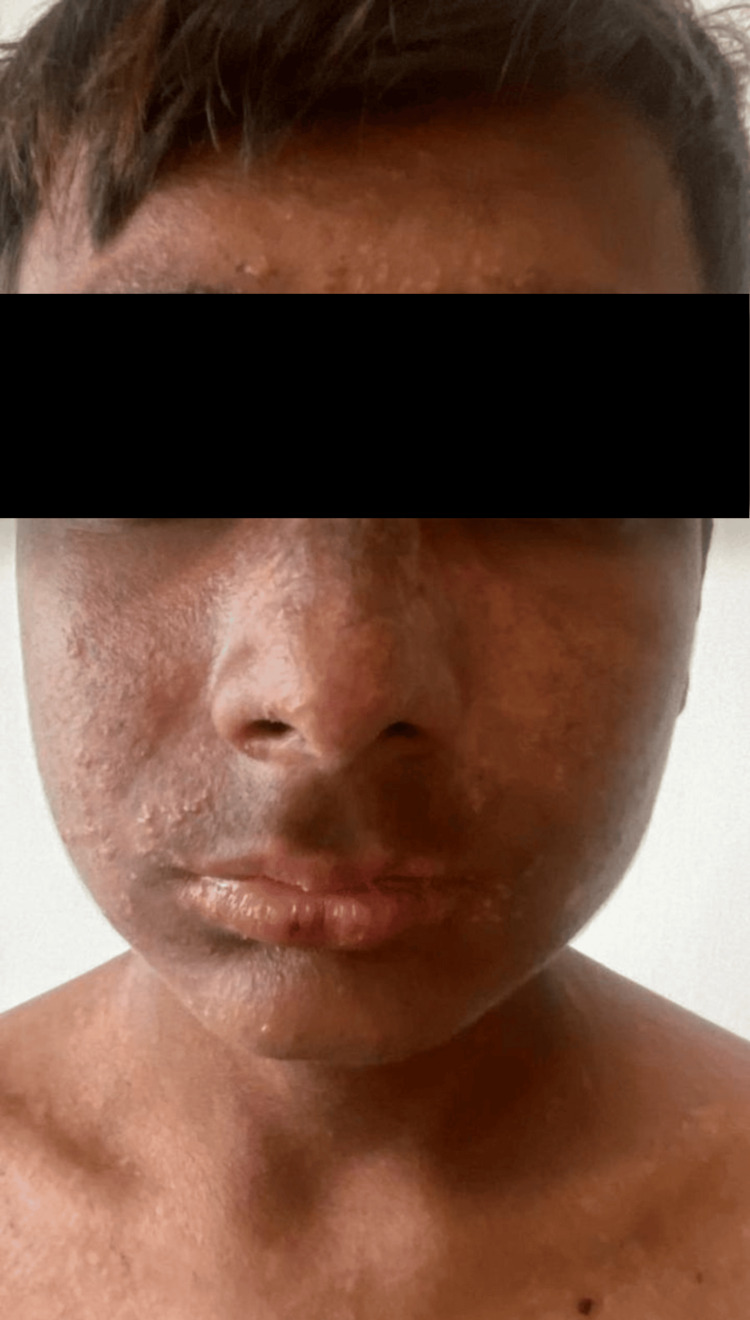
Multiple small vesicles and papules on the cheeks and forehead. A few crusted erosions are present on the lower lip, along with hyperpigmentation of the nose and perioral region.

**Figure 2:**
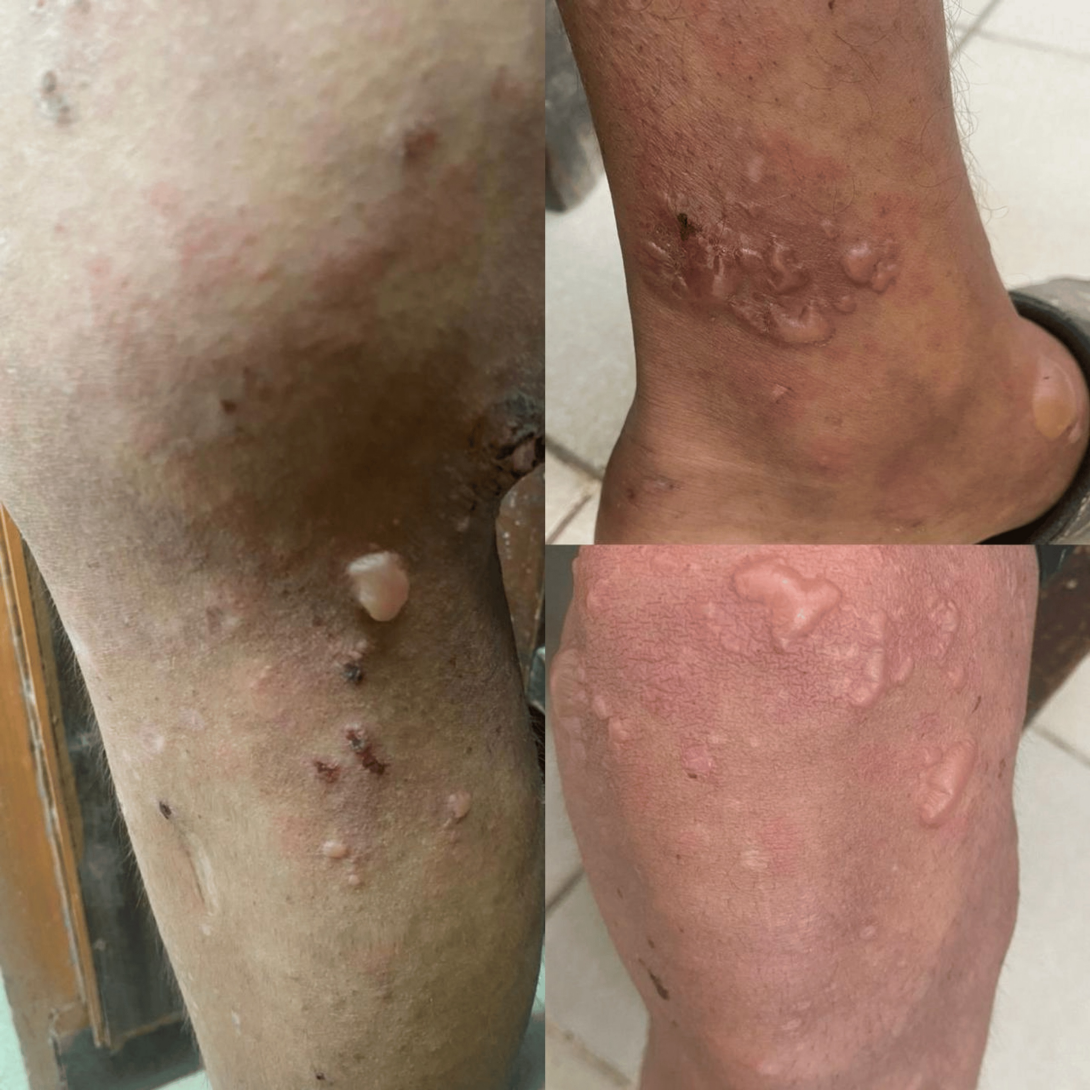
Multiple tense, clear-fluid-filled bullae of varying sizes over the lower legs and ankles, arising on an erythematous background, with scattered papules, excoriations, and post-inflammatory hyperpigmentation.

**Figure 3:**
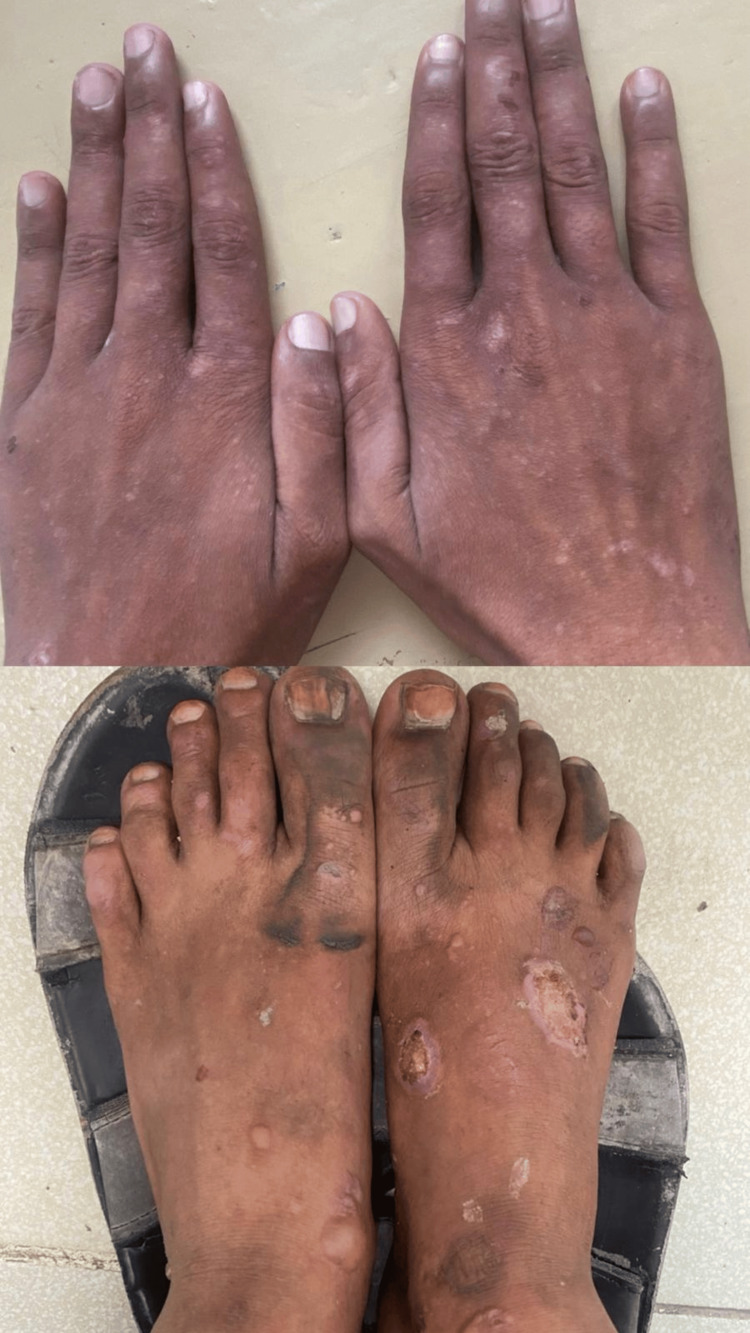
Multiple tense bullae and vesicles on the dorsum of both feet, some ruptured, forming crusted erosions. Post-inflammatory hyperpigmentation and depigmented macules are present on both hands and feet.

A 14-year-old male patient presented to the dermatology outpatient department of Dr. Ruth K. M. Pfau, Civil Hospital Karachi, Pakistan, with a complaint of recurrent blistering lesions involving the body for one year. Initially, he developed small, fluid-filled blisters over the trunk, which gradually progressed to involve the face, extremities, palms, and mucosal surfaces (Figure 1, Figure 2, Figure 3).

**Figure 4:**
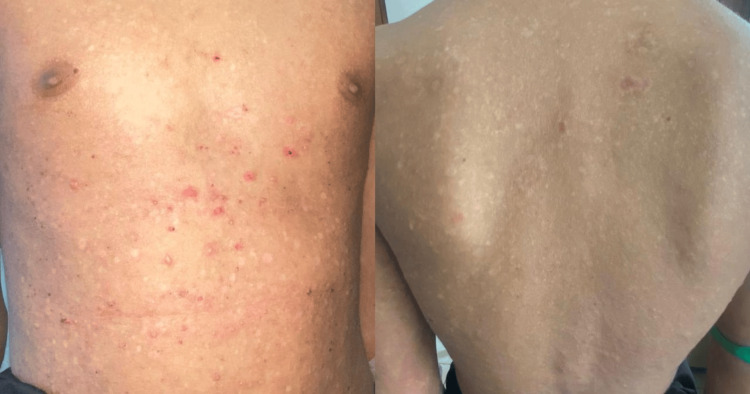
Multiple discrete erythematous to skin-colored papules and vesicles scattered over the anterior chest, abdomen, and back. Some excoriation marks and crusted erosions are present.

The blisters were tense, pruritic, and variable in size, often enlarging over time. They ruptured spontaneously after several weeks, discharging clear fluid and healing with crusting and post-inflammatory hyperpigmentation or hypopigmentation (Figure 4).

**Figure 5:**
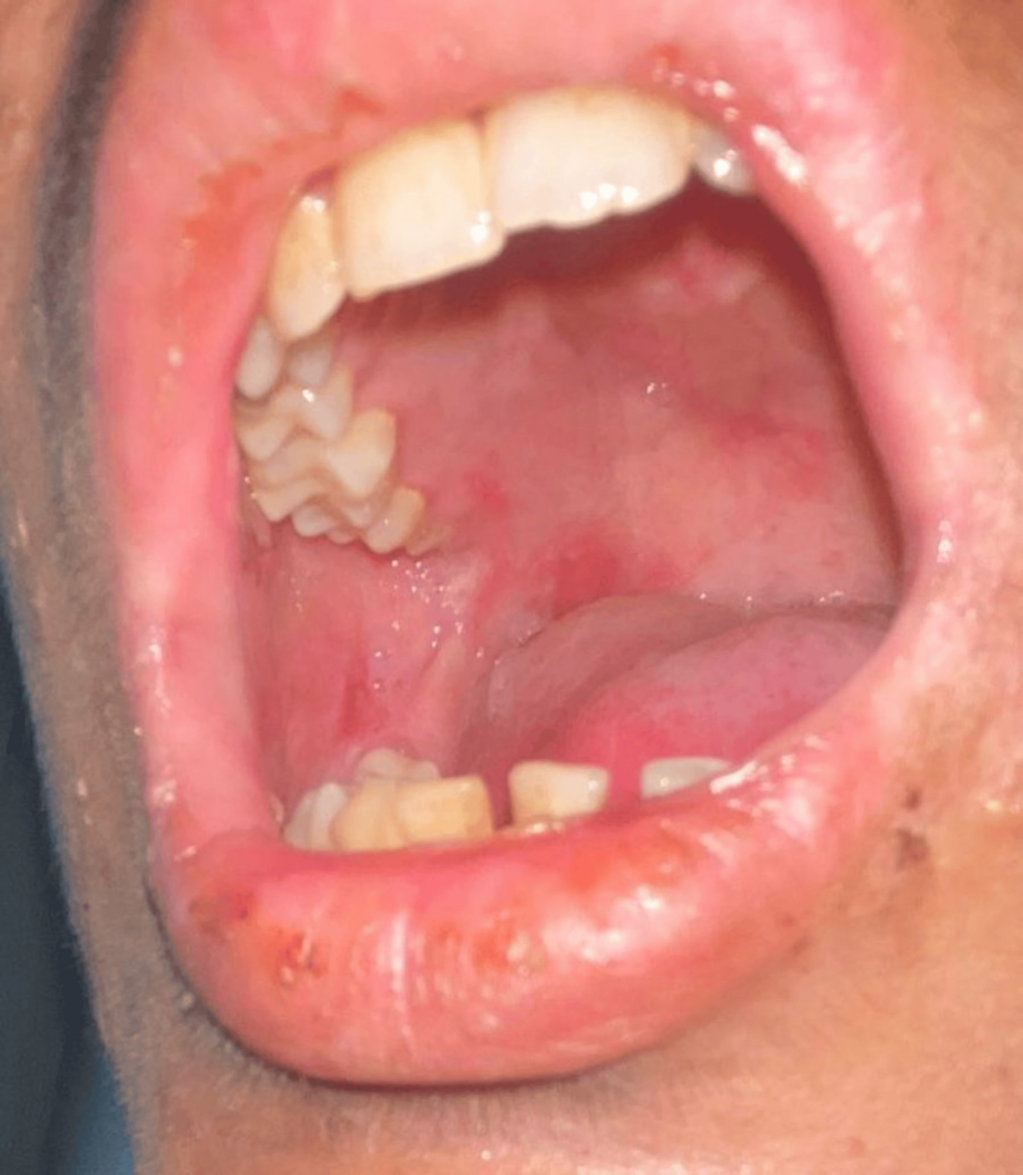
Oral cavity showing multiple ulcerations on the hard palate and buccal mucosa, with crusted erosions on the borders of the lips.

Oral and genital mucosa were markedly involved, with painful erosions resulting in difficulty eating and drinking (Figure 5). The lesions showed partial improvement with topical therapies prescribed by local practitioners.

Approximately four months after the onset of cutaneous manifestations, the patient developed gradually progressive facial and pedal edema, more pronounced in the mornings, which later progressed to generalized body swelling. This was accompanied by decreased urinary frequency, dysuria, and intermittent hematuria, prompting admission to a tertiary care nephrology center for further evaluation.

Review of systems revealed photosensitivity for the past three years, with worsening of cutaneous lesions upon sun exposure, along with intermittent low-grade, undocumented fever, fatigue, diffuse hair loss, recurrent oral ulcers, xerostomia, and inflammatory small-joint pain associated with mild morning stiffness. There was no history of Raynaud phenomenon. Review of the gastrointestinal, respiratory, and central nervous systems was unremarkable. There was no family history of similar dermatologic conditions, and the patient’s growth and developmental history were appropriate for age.

Given the strong autoimmune history, the patient was evaluated for SLE with renal involvement. Laboratory evaluation revealed anemia, hypoalbuminemia, proteinuria, and a positive antinuclear antibody (ANA) test with low complement levels. The laboratory investigations are summarized in Table 1.

**Table 1:** Laboratory investigations of the patient ANA, antinuclear antibody; anti-dsDNA, anti-double-stranded DNA; C3, complement 3; C4, complement 4; ESR, erythrocyte sedimentation rate

Test	Value	Normal range
Hemoglobin	12.3 g/dL	11.5-15.5 g/dL
White blood cell count	8.06 × 10⁹/L	5.0-13.0 × 10⁹/L
Platelets	261 × 10⁹/L	150-450 × 10⁹/L
Urea	16 mg/dL	10-40 mg/dL
Creatinine	0.52 mg/dL	0.3-0.7 mg/dL
Sodium	142 mmol/L	135-145 mmol/L
Potassium	3.1 mmol/L	3.5-5.1 mmol/L
Chloride	106 mmol/L	98-107 mmol/L
Bicarbonate	25 mmol/L	22-28 mmol/L
Total bilirubin	0.19 mg/dL	0.1-1.0 mg/dL
Alanine transaminase	27 U/L	<45 U/L
Aspartate transaminase	29 U/L	<45 U/L
Alkaline phosphatase	129 U/L	150-420 U/L
Albumin	1.57 g/dL	3.5-5.0 g/dL
ESR	110 mm/hr	<10 mm/hr
Urine detailed report	Albumin ++++	
	Red blood cells - occasional	
ANA	++++ Homogeneous, 1:320	
Anti-dsDNA	Negative	
C3	0.58 g/L	0.89-1.7 g/L
C4	0.10 g/L	0.16-0.3 g/L

**Figure 6:**
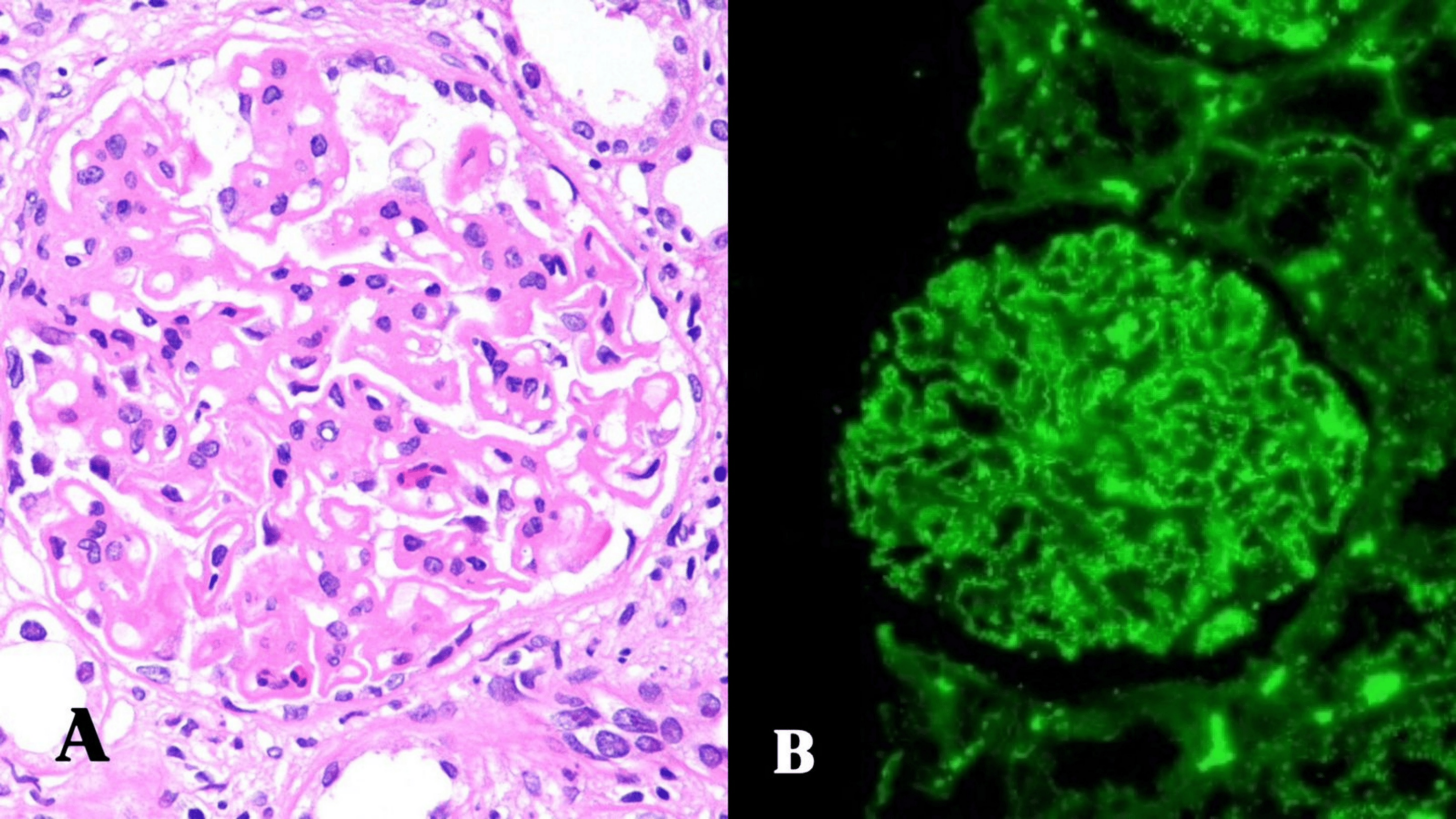
(A) High-power view showing one glomerulus with diffuse thickening of GBMs. No proliferation is seen (H&E, ×400). (B) IF showing diffuse, granular, membranous positivity of IgG along capillary walls (IF for IgG, ×400). GBM, glomerular basement membrane; IF, immunofluorescence

Histopathological examination revealed subepidermal blister formation with a predominantly neutrophilic infiltrate. DIF demonstrated linear deposition of immunoglobulins (IgG, IgA, and IgM) and complement component C3 along the basement membrane zone (BMZ). A renal biopsy was performed, revealing features consistent with membranous glomerulonephritis: lupus nephritis grade V. Immunofluorescence showed strong IgG positivity along capillary walls and was negative for IgA, IgM, and C1q (Figure 6).

The patient was started on oral prednisolone at 0.5 mg/kg/day and low-dose dapsone at 0.5 mg/kg/day to control the skin lesions. Baseline glucose-6-phosphate dehydrogenase (G6PD) levels were measured before the initiation of dapsone and were found to be normal. Topical antibiotics and corticosteroid creams were applied to affected areas, and sun protection was advised. The patient showed a good initial response, with marked improvement of the lesions; however, new lesions appeared on follow-up. Hydroxychloroquine at 6 mg/kg/day was then added to help manage the cutaneous disease.

Because of ongoing renal involvement, mycophenolate mofetil was started at 500 mg/m² twice daily, with gradual tapering of steroids. At two months, laboratory tests showed progressive improvement, including complete resolution of proteinuria and normalization of complement levels, indicating a positive renal and immunological response. The plan is to continue mycophenolate mofetil for six months, followed by transition to azathioprine for maintenance therapy. The patient remains clinically stable and continues long-term follow-up with both dermatology and nephrology services.

## Discussion

BSLE is a rare cutaneous manifestation of SLE that presents as an acute, tense vesiculobullous eruption involving both sun-exposed and sun-protected areas. Commonly affected sites include the face, neck, upper abdomen, axillae, proximal limbs, and mucous membranes [5]. The lesions often progress to erosions and typically heal without scarring, although pigmentary changes have been documented [4]. Patients with BSLE frequently exhibit extracutaneous involvement, most notably arthralgia, arthritis, cytopenias, and lupus nephritis, while serositis and neuropsychiatric manifestations are seen less commonly [4].

The disease is driven by autoantibodies targeting the noncollagenous domains 1 and 2 (NC1 and NC2) of type VII collagen, which is the major anchoring fibril component located in the sublamina densa of the BMZ. These autoantibodies, after binding to type VII collagen, form immune complexes along the dermoepidermal junction, leading to linear or granular deposition of IgG, IgA, and complement (C3) on DIF [4]. Complement deposition triggers the recruitment of inflammatory cells, including neutrophils, which accumulate at the dermal papillae and along the BMZ, causing inflammation and disruption of the anchoring fibrils, leading to subepidermal blister formation with a dense neutrophilic infiltrate. However, similar histopathological findings can also be seen in epidermolysis bullosa acquisita and dermatitis herpetiformis. Additional autoantibodies, including those directed against BPAg1, laminin 5, laminin 6, and BPAg2, have also been identified [6].

A variety of medications have been implicated as triggers for BSLE, including tumor necrosis factor-alpha inhibitors, IL-6 inhibitors, terbinafine, methimazole, and penicillamine [7]. Notably, a recent report by Lesort et al. described two patients who developed BSLE flares after their first dose of anifrolumab, an interferon alpha receptor 1 antagonist approved for moderate to severe SLE [8]. In our patient, however, no identifiable trigger, including medication, was found.

Diagnosing BSLE can be challenging due to its resemblance to other blistering disorders such as bullous pemphigoid, pemphigus vulgaris, dermatitis herpetiformis, epidermolysis bullosa acquisita, linear IgA bullous dermatosis, porphyria cutanea tarda, Stevens-Johnson syndrome, and toxic epidermal necrolysis [9]. ANA is typically positive given the underlying SLE [4], and patients may also have anti-dsDNA, anti-Smith, anti-Ro (SSA), anti-La (SSB), and anticardiolipin antibodies [4]. Skin biopsy plays a crucial role in differentiation, showing a subepidermal blister with dense neutrophilic infiltration in the upper dermis [6]. DIF reveals deposition of IgG, IgM, IgA, and C3 at the BMZ in lesional and perilesional skin [9], while on salt-split skin indirect immunofluorescence, antibodies bind to the dermal side.

Dapsone is considered the first-line treatment for BSLE, achieving a response in approximately 90% of cases, provided the patient has normal G6PD levels [9]. For those unresponsive to dapsone or with severe systemic involvement, systemic corticosteroids are added [10]. Second-line options include steroid-sparing immunosuppressants such as azathioprine or mycophenolate mofetil and biologics such as rituximab (anti-CD20). Donelli C. reported successful use of belimumab, an IgG1λ monoclonal antibody targeting B-lymphocyte stimulator, in a patient with mucocutaneous BSLE [10]. Belimumab, initially approved by the FDA for severe SLE, has also shown efficacy in cutaneous lupus erythematosus and BSLE [11].

Our case underscores the importance of considering BSLE even in male patients presenting with cutaneous blisters alongside systemic features, as in our patient, who had renal and articular involvement. This case contributes to the limited literature on BSLE in male patients from South Asia, particularly Pakistan.

However, the report is limited by its nature as a single case study, which restricts the generalizability of the findings to broader patient groups. Additionally, the diagnosis of BSLE can be challenging because of its close mimics, such as epidermolysis bullosa acquisita, bullous pemphigoid, and dermatitis herpetiformis. Hence, it requires specialized laboratory and histopathological investigations, such as antibody screening, DIF, and salt-split testing. In resource-limited settings such as Pakistan, access to these advanced diagnostic tools may be limited, which can delay or complicate accurate diagnosis.

## Conclusions

This case highlights the importance of recognizing BSLE as a rare but distinct manifestation of SLE that can occur in male patients, contrary to its usual predilection for women. The presence of acute vesiculobullous lesions in a patient with systemic features such as arthritis and nephritis should prompt consideration of BSLE, even in populations and genders in which it is less commonly reported. Early diagnosis, supported by histopathology, immunofluorescence, and serological markers, is critical for appropriate management. Our case contributes to the limited body of literature documenting BSLE in males from South Asia and emphasizes the need for heightened clinical awareness of this entity to ensure timely treatment and prevent complications.
